# Introduction of a Single Electronic Health Record for Maternity Units in Ireland: Outline of the Experiences of the Project Management Team

**DOI:** 10.2196/38938

**Published:** 2023-05-12

**Authors:** Orla Maria Sheehan, Richard Anthony Greene, Joye McKernan, Brendan Murphy, Caroline Cahill, Brian Cleary, Fiona Lawlor, Michael Robson, MN-CMS National Project Team

**Affiliations:** 1 Department of Obstetrics and Gynaecology University College Cork Cork Ireland; 2 Department of Obstetrics and Gynaecology National Perinatal Epidemiology Centre University College Cork Cork Ireland; 3 Department of Paediatrics and Child Health University College Cork Cork Ireland; 4 Maternal and Newborn Clinical Management System Health Service Executive Dublin Ireland; 5 Department of Obstetrics National Maternity Hospital Holles Street Dublin Ireland

**Keywords:** baby, babies, data management, data quality, electronic health record, health management, implementation, information management, Ireland, lessons learned, management system, maternity, maternal, mother, newborn, optimization, planning, pregnant, pregnancy, project management

## Abstract

Electronic health records (EHRs) are being introduced worldwide. The change from paper to electronic records has not always been a seamless or quick process; however, EHRs are viewed as central to updating modern health care, especially organization structures and delivery of sustainable care with the potential for joint decision-making with the patient. The objective of this viewpoint paper is to outline how an EHR is being developed in Ireland. The focus of the Maternal & Newborn Clinical Management System project is the design and implementation of an EHR for all women and babies in the maternity services in the Republic of Ireland. The paper also outlines the lessons learned from the planning to the optimization stage of the project. The paper was developed through discussions with the project management team and their completed reports that outline the lessons they acquired from each project stage. Key lessons learned from each stage of the project are highlighted. This viewpoint paper explains how the national project management team is implementing the EHR and outlines the experiences and lessons learned and the challenges ahead following the phase one introduction. The Maternal & Newborn Clinical Management System is an example of a clinician-led, patient-focused, change management project from its inception to implementation. The introduction of EHRs is essential in modernizing health care and optimizing patient outcomes through the accurate and appropriate use of data.

## Introduction

Electronic health records (EHRs) are essential in attempting to update modern health care [1] and are being introduced across Europe, North America, Australasia, and the Middle East [2]. The change from paper to electronic records has been described as complex [3], particularly in the hospital setting compared to private industry, due to the nature of the organizational setting that involves caring and curing, data security, confidentiality, and data accuracy [4]. Many complex steps have been identified in the implementation of an EHR from procurement, design, building, testing, and training through to adoption and optimization [5]. A hospital-wide EHR implementation needs to consider a number of organizational, human, and technological factors as well as organizational structure, culture, technical infrastructure, financial resources, and coordination [4,5].

The need for digital information and connection has evolved in the last 2 decades [2] and in particular since the COVID-19 pandemic with a reliance on the digital world to connect. Over half the world’s population (60.9%) use the internet and two-thirds of the world’s population (66.9%) have a mobile phone [6]. In Ireland, it is estimated that 3.68 million (73.5% of the population) had a smartphone in 2021 with a projected 3.8 million by 2024 [7,8]. The delivery of safe and effective health care is dependent on the availability of accurate and up-to-date data in a legible, reliable, and timely manner [9]. It is through the use of an EHR that health data can be available in the correct place at the correct time to enable timely clinical decision-making.

### Maternal & Newborn Clinical Management System

**Figure 1:**
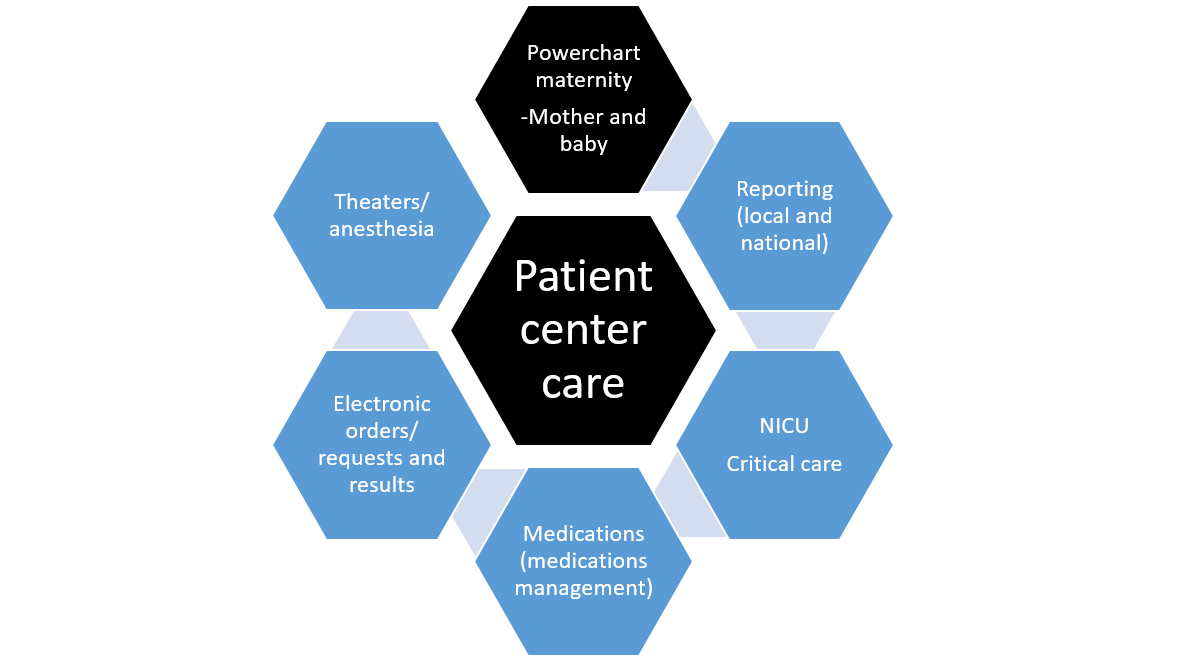
Connected elements of the MN-CMS project including Maternity, neonatal intensive care unit (NICU), Medications and Reporting. MN-CMS: Maternal & Newborn Clinical Management System.

The Maternal & Newborn Clinical Management System (MN-CMS) pathfinder project is the design and implementation of an EHR for all women and babies in the maternity and gynecology services in the Republic of Ireland [10]. The MN-CMS is a significant development in health care, the first electronic national system in maternity and newborn care anywhere in the world. The introduction of the MN-CMS is a necessary step in modernizing health care and it aligns with the strategic goals of the Department of Health and the eHealth Ireland digital roadmap [11]. At an international level, this national EHR implementation is the first of its kind as no other country has achieved a fully interoperable national EHR within the community or acute care settings [5,12,13]. The MN-CMS includes all the relevant processes required including order communication, medications, anesthesia, and theater as well as all clinical documentation for maternity, newborn, and gynecology (see Figure 1). Laboratory orders are a closed loop of order communication from initial order placement to laboratory results and clinical endorsement and review of results. Medications are fully electronically prescribed with assisted decision support and electronic recording of administration. The MN-CMS has the potential to design the most cost-effective models of care and establish a national epidemiological database [14], which will inform national clinical care, audit, research, and business intelligence.

As of 2016, the Republic of Ireland had a population of 4,761,865 [15] (with an estimated 5.01 million in April 2021 [8]) with an average of 65,000 births a year [15]. The maternity units in Ireland vary in size: The smallest unit has about 1000 deliveries per year, and the largest unit has over 9500 deliveries [16]. The information and communication technology (ICT) systems across the units vary. Some units use only paper charts, while other units have limited electronic systems. General practitioners, who provide a shared-care model of antenatal care, in Ireland, use electronic medical records; however, the system was not linked to the national hospital system. With the introduction of the MN-CMS, the pregnancy and birth records for pregnant women and their infants are now linked to the general practitioner systems with MN-CMS through a messaging broker system called *Healthlink*. As with the development of all EHRs, this project is a major change project not an ICT project; it involves multiple changes across all aspects of maternity services at the individual level through to the organization level. This project involves transitioning all maternity units over time to 1 single EHR. Keeping the mother and baby at the center was the primary principle set out by the project board, and it was envisioned that the MN-CMS would record practice and not decide practice [2].

### Aim

The aim of this paper is to describe the key observations and lessons learned from the national project team implementing the MN-CMS.

## Methods

### Overview

The methods used to develop these valuable outcomes were through discussions with the project team, post go-live workshops focused on lessons learned, and the phase one closure report developed by the national project team in conjunction with key stakeholders. Following each hospital’s go-live phase, a lessons-learned workshop was facilitated by the national project team with an approach of categorizing learnings through people, process, and technology. Each workshop had representation from key stakeholders from hospital management, project managers, to end users of the system. Clinicians including midwives, doctors, nurses, health and social care professionals, project managers all participated in interactive workshops which collated all feedback and lessons into the categories of people, processes, or technology. From all this feedback, a phase one closure report was developed detailing the key lessons and experiences of the project team in implementing MN-CMS. This paper describes the feedback from the workshops, discussion with the project team, and the phase one closure report categorizing the learnings in the various stages of the project, which include planning, implementation, and optimization.

### The Planning Phase

The origins of the project were the establishment of the MN-CMS project board whose aim was to establish a single record and a single source of truth for health-related data for all women and their babies in Ireland [2,14]. The board had oversight to procure and implement the EHR [14] and included stakeholders from the Institute of Obstetrics and Gynecology, the Faculty of Paediatrics, nursing and midwifery, pharmacists, and representatives from the Department of Health and Healthcare ICT [2,14]. Further stakeholders such as general practitioners and anesthetists were included as the project progressed. The establishment of the project board marked the beginning of a change management project across the maternity services in the Republic of Ireland [2]. A patient representative was not included and should have been considered at this stage.

A need-based assessment was developed to establish a design specification, and this was carried out by the project board members and senior health care managers [2,14]. The assessment was in consultation with the 19 maternity units in Ireland [14]. Following the design specification, the public procurement process commenced in 2011 with contracts signed in 2014 [14]. The preferred vendor following public procurement was *Cerner* [2,14] (more recently acquired by Oracle and now referred to as *Oracle Cerner*). Finally, the MN-CMS team initiated the implementation program in 2014 [14]. This procurement process was time consuming, and strong leadership was essential when planning and consolidating scope and deciding on the preferred software vendor.

The MN-CMS national board had responsibility for ensuring a governance structure was in place [2,14]. This guaranteed a successful deployment of the EHR to the maternity service [2,14]. The deployment of phase one was with 4 units (2 stand-alone units and 2 colocated units) from 2016 and will continue on a phased basis [14] based on the availability of public funding from the relevant government departments [2,14]. A strong governance structure is a requirement for the success of a project.

Once the vendor had been appointed, numerous work streams were established for each clinical area such as maternity, neonatal, medications, health and social care professionals, order communications, clinical reporting, and research [14]. Representation was sought from each of the 19 maternity units in the country [14]. Each work stream developed multiple workflows based on their specific delivery of care such as the maternity booking visit, return visits, admissions, labor, and delivery, elective and emergency cesarean section, stillbirth and neonatal death, blood transfusion, neonatal intensive care unit (NICU) admission and discharge [2,14]. One NICU work stream was established, however, in hindsight; this should have been a NICU or newborn work stream based on the patient rather than any particular health care professional, as it led to different views and data entry for each health professional caring for well babies and NICU babies. A key to design and development was to sit down with multidisciplinary team experts and map out all current workflows; then design the new workflows with input from the system specialists. Adequate representation is essential for workflow development. Crossover of work streams is necessary, for example, NICU or newborn leading to 1 baby chart design rather than different views for postnatal midwife and neonatal staff. Key lessons from the planning phase are outlined in Textbox 1.

**Lessons learned from the planning phase of the Maternal & Newborn Clinical Management System (MN-CMS) implementation.**

**Planning Phase Lessons**

- Patient representative at an early stage
- Strong leadership needed for initial procurement
- Strong governance structure required throughout the project
- Involve the clinical teams and experts in workflow development

### The Implementation Stage

The responsibility of the phase one implementation rested with the implementation team that was established in 2014 [2,14]. This project team initially consisted of a small team with clinicians at the center of this team [2,14]. Over time, the scale of the EHR implementation challenge highlighted that additional resources were required [14], and that clinical staff were instrumental in all elements of the project [14]. An adequately resourced project team is essential, and a realistic expectation of the commitment and responsibility expected should be clearly defined for those involved.

**Figure 2:**
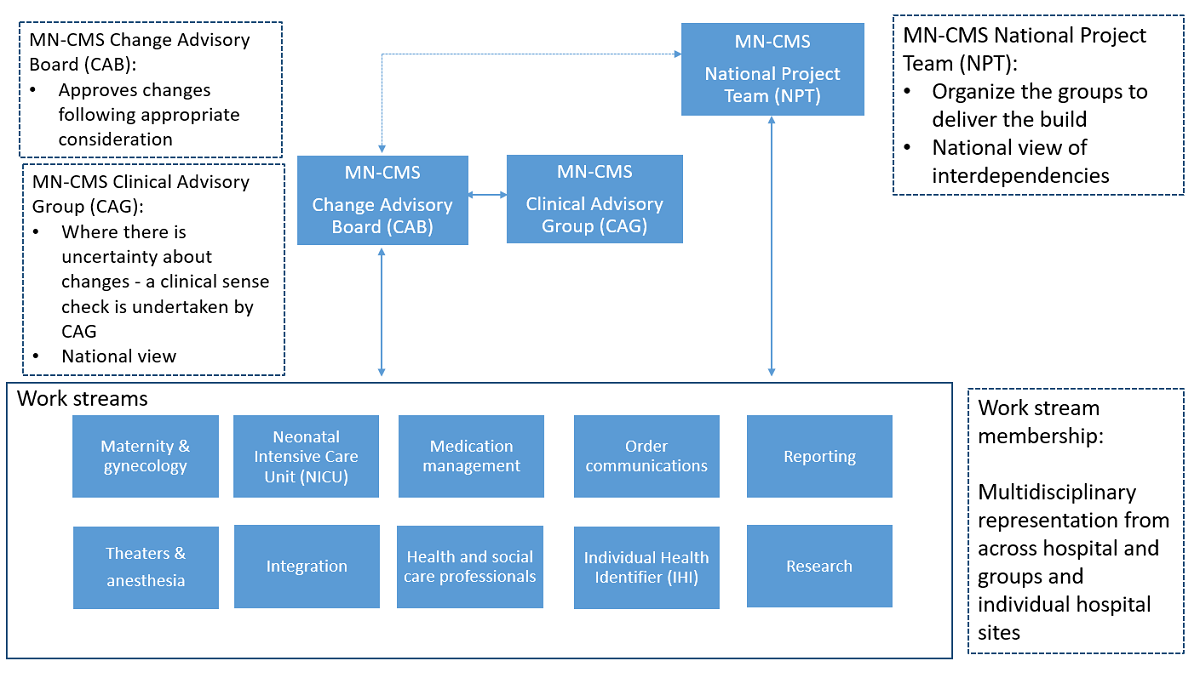
Outline of work streams required for MN-CMS project. MN-CMS: Maternal & Newborn Clinical Management System.

The previously mentioned clinical work streams conducted the current state analysis by working with the national project team and the vendor. This current state analysis mapped the current workflows of all the hospitals in the phase one sites [2,14]. This involved mapping the patient journey from the first referral to the hospital for care right through the discharge process following delivery for both mother and baby [14]. Between the maternity and NICU work streams, approximately 54 workflows on average were mapped for each hospital [14]. This process required relevant clinical personnel familiar with their own area and delivery of care and a functional structure to achieve this. Figure 2 outlines this structure.

The future state analysis was developed from the current state workflows [14]. This process transformed the patient journey into the new electronic environment of future state workflows that would be the foundation of design and build for the EHR [2,14]. The future state analysis is the key foundation for the design and building of the system, and gaining consensus across all sites is beneficial.

The design and build phase involved a collaborative approach across the national project team, the vendor, and the work streams [14]. This phase of the project was challenging due to the complexity involved in designing and configuring a comprehensive EHR [2,14]; the work streams had representatives from all 19 units. This complexity of this work was underestimated by all involved [14]. The recommendations made were fed back to the national team and the vendor [14]. Inclusion from the broader work stream group is necessary to assist buy-in to the project. However, a smaller group, comprised of clinicians and subject matter experts, made final decisions on the design, and the vendor then completed the configuration and build [14].

The purpose of the future state validation was for the MN-CMS team and work stream members to present the system with the final design and build to those working in the maternity services [14]. It was an opportunity for attendees to gain an understanding of how it would work in their hospital by demonstrating various patient journeys and workflows [14] and allowed them to get some hands-on experience with the system [2,14]. The future state validation needs to reach as many people as possible, and champions from individual hospitals need to be engaged with the process.

In terms of changes to the system, there is a strict change control protocol in place since the completion of the design and build phase [2,14]. A requested change to the system may have significant knock-on effects on individual workflows or indeed individual units. To this end, weekly meetings are held to manage and review change requests by the Change Advisory Board [14]. Requested changes in the system are assessed individually as 1 change for 1 unit may not be acceptable in another, all changes need to be agreed by all units, and all work streams affected before being implemented [14] in the live production environment, maintaining a national core. Changes are first implemented and tested in a test environment before progressing to production. A change control policy needs to be strict and adhered to, throughout the process. The impact on the workflow and training needs to be assessed with each change. A test or nonproduction environment is necessary to test the change prior to implementation in the live system.

The testing phase consisted of 2 phases—system and integration testing [14]. Test scripts were created, by clinicians, based on the clinical workflows and the various scenarios that might occur for both mother and baby [14]. The system testing phase involved testing the system against the workflows to ensure the design was functioning as expected [2,14]. Integration testing consisted of testing the systems with all other third-party systems (eg, patient administration system and laboratory information systems) [14]. This testing was undertaken at each individual site based on different patient administration and laboratory information systems and to validate their workflows [14]. Integration testing also included the testing of printers, wristbands, and barcode scanners [14]. Device integration with the EHR for the neonatal vital signs monitors and ventilators and the fetal monitoring (cardiotocograph-monitoring) solution—FetaLink (FetaLink provides a graphical display of the relationship between fetal heart rates and contraction data in the EHR. It displays waveforms and annotations, which can be viewed in real time by care providers in inpatient or outpatient settings [2]) were all tested during this testing cycle [14]. These devices were tested using demo modes and simulations. The Downtime System Access Viewer (724) used to view the patient chart in the event of a planned or unplanned downtime also had to be tested at each site [14]. All test issues were logged to a web portal accessed by the project team and vendor. Each test issue was prioritized, fixed, and retested and then closed by the clinical team of testers [2]. Testing takes time with a large team. The allocated team in this project was too small. The personnel involved in testing need to be subject experts and expert users with on-the-ground clinical knowledge of the workflows and processes in each site.

The first steps prior to the deployment of MN-CMS were a Wi-Fi connectivity survey of each site, and an assessment of the extra power points, data cables, and ports required for the new hardware was installed in each unit [14]. The additional infrastructure required to implement MN-CMS included computers on wheels, desktop computers, electronic whiteboards, printers, scanners, and barcode readers [14]. The purchase of these items followed the national procurement process and was a multidisciplinary exercise involving ICT, biomedical engineers, end users, and infection control teams [2,14]. Local ICT teams are essential and need to feel part of the project, and issues arising need to be solved as a single local EHR team. Ongoing support by local ICT of hardware devices and connectivity is essential.

Approximately 2500 staff benefited from training, across the 4 phase one sites [14]. Classroom-based training consisted of 1-3 days duration depending on their system role [14]. Training course plans were prepared based on patient journey workflows, the train domain was populated, and following a train-the-trainer program training was provided to staff in each hospital by their peers [14]. These trainers initially provided intense training to a group of superusers (both trainers and superusers were an invaluable support for the early weeks of go-live to support colleagues in each clinical area [2,14]). The trainers localized their training plans for their specific hospitals and kept detailed training records to ensure that all staff attended training [14]. Logistic planning was essential to schedule the training and the backfilling in clinical areas as staff were released to train. Personnel need to be identified to deliver training and support to medical, midwifery, nursing, other health care professionals, and administration disciplines. These are vital roles, and some will be required after go-live. Providing a play domain (not available in this project) for staff post training would be beneficial—staff need to build their confidence through familiarization and practice.

The go-live phase involved setting up of user accounts, deployment of passwords, data migration, and the staffing of a 24-hour, 7-day-a-week command center to support staff over the go-live weekend and the post go-live early live support period. Each go-live was supported on the ground by the members of the National Project Team, the vendor, the team of engineers and solution specialists, project managers, and staff from the other phase one sites [2,14]. Data migration began in the weeks coming up to go-live for all patients close to term (end of pregnancy) and for inpatients (women and babies) who were expected to remain for a period after go-live. If a woman delivered and was documented on paper before go-live, they remained on paper. Patients who were attended in labor after the go-live time had delivery documentation on the EHR and completed their care on the EHR with appropriate agreed data documented including past history, allergies, medications, and risk factors [2]. There was a “wash-through” period before all paper records were removed [2]. The go-live phase was a big bang, done over a weekend to allow a phased introduction of the EHR to inpatient care, then outpatient care after the weekend [2]. All ward managers should be trained as superusers, this ensures they can feel confident in supporting their ward staff over go-live. This was not achieved for our first go-live, and the deficit was quickly recognized and corrected for future go-lives.

The go-live phase is the focus of the project from inception—it is the prize for the project team and the individual service. This is necessary to achieve the target worked on in the project. However, the local project team or an equivalent team of subject experts and expert users’ needs to be in place before go-live and ready to carry on support and optimization through training, assessment, and development of workflows and associated documentation and identifying and getting fixes for identified risks in the new world of digital documentation. A clear transition team and a plan to assist with the adoption and optimization of the new business model are needed before the go-live phase.

The implementation of electronic medicines management within an EHR offers the potential to streamline patient care and to engineer safe medication use processes [2]. Currently implemented within MN-CMS are clinical decision support functionality including allergy checking, interaction checking, dose range checking, customized enterprise rules, weight-based dosing, prewritten order sentences, and care plans [2,14]. Electronic prescribing has been demonstrated to promote safe and effective prescribing practices and to reduce the risk of errors [2,14]. The system facilitates clinical pharmacy services which have been demonstrated to improve patient outcomes and reduce the risk of serious patient harm [14]. The benefits of getting the medication management right will have implications for patient safety; however, it does need to be resourced adequately through funding and staff. Key lessons from the implementation stage are outlined in Textbox 2.

**The implementation phase of a lesson learned.**

**Implementation stage lessons**

- Adequately resourced project team and clarity of the commitment needed is essential
- Relevant clinical personnel to clearly outline workflows
- The Future state analysis is the key foundation for the design and build
- Design involves a wide team, but some final design decisions were made by a smaller group
- The Future state validation needs to reach as many as possible to fully engage with the process
- Strict change control process and approval is essential
- Testing requires a large team of clinical users and subject matter experts
- Local information and communication technology involvement is essential
- Peer trainers need to be engaged early and these vital roles are required post go-live in the support phase
- All ward midwife or nurse managers should be trained as superusers ensuring they are confident to support their staff over the go-live period
- A transition team is needed post go-live to assist with the adoption and optimization of the new business model
- Medication management benefits can have positive implications for patient safety, but it does need to be resourced adequately

### Optimization Phase

The medication management element of the EHR was developed by the MN-CMS project team including clear order sentences with correct formulation dose [2]. Some medication errors were highlighted early post go-live as a key area that could be improved to enhance patient care. Enhancements such as the development of care plans, weight-adjusted dosing among others led to safer prescribing [2]. The EHR allows enhanced assessment of medication error, and while this takes resources and time and subsequent staff training it is an essential element toward better patient safety.

The reductions in documentation time should allow for better records [2,14]. Staff can now avoid duplicating information (as they did on paper); an example is developing the baby chart transcribing information from the mother’s chart—an instant transfer of agreed information saves time [2]. Easy access to records allows for all telephone consultations to be easily and clearly documented as part of the record [2,14]. Ongoing staff training is required to fully assist in the efficiency of their documentation [14].

In terms of data quality, routine data collection needs to be simple, clearly defined and an integral part of normal care, and the responsibility of all health care staff [2,14]. The need for high-quality and accurate data was recognized early in conjunction with European Union directives [17]. Data quality personnel were put in place to check for data errors based on output reports [2,14]. Local information governance teams were set up to ensure the integrity of the data. The National Project team also has an Information Governance Group to deal with issues on a national basis for a single national system [2,14].

The MN-CMS now has the capability of producing clinical reports for audit, research, financial, and management requirements. These reports have taken time to build and test; however, they will be an invaluable data source in the future [2,14]. The reporting function has highlighted data quality issues related to poor data entry. Mitigation has been put in place; processes including the employment of data quality staff, daily data quality checks that highlight where issues arose from these checks, and staff are then contacted and requested to complete the data they have omitted [2,14]. Based on normal care documentation, Ireland’s maternity and neonatal service will have a high-quality database to assess the quality of that care [2]. Demographic information for all patients will be easily accessible, and data that would not have been available before will now be available [2]. Routine data collection needs to be simple, clearly defined, and an integral part of normal care and the responsibility of all health care staff [2]. Detailed consideration of the data elements required in the planning and configuration is vital and proved valuable in this project. Ensuring staff know their responsibility for data quality when entering data needs to be part of their training. Data quality assessment is required and appropriate responses including identification of errors to staff, further training for staff, and changes in configuration as required.

Patient involvement is necessary, and the project worked with the Women’s Council of Ireland to develop women’s focus groups to discuss the development of the EHR and take advice from patients [14]. One of the interesting lessons from this engagement was the belief that we had already digitized the care documentation—we were behind the public expectation. Patient access to their information was considered very important. MyHealthPortal will be a national web-based site designed for use by patients and their caregivers [2,11,14]. Its purpose is to engage patients in self-care, joint decision-making and empower them to take a more active role in their health care management [2,11,14]. This element of the MN-CMS project has not been set up to date. Prior to the EHR for the duration of their pregnancy, women did have access to their paper medical charts. The MN-CMS board is committed to ensuring women have this access again [14]. This element is key as internationally, as there is an ambition toward providing patient-accessible EHRs [2,18,19]. There are, however, some limiting factors that include security and privacy concerns, legal constraints, and low uptake of other web-based resources for patients [20]. Engagement with patient representatives early in the process is necessary. As go-live is approaching inform patients during their visits of the expected changes and potential disruption as the hospital transitions to an electronic-based record for their care.

MN-CMS trainers are an invaluable support to the local project teams and to the end users on the ground. Since go-live, the 4 sites have engaged with their end users in optimization sessions where new, advanced, and updated functionality has been taught. There is always ongoing, or business-as-usual training of new staff and locum doctors being carried out throughout the year [14]. Keeping the initial trainers and superusers involved throughout the process will lead to them being champions and optimization agents, in the future.

**Figure 3:**
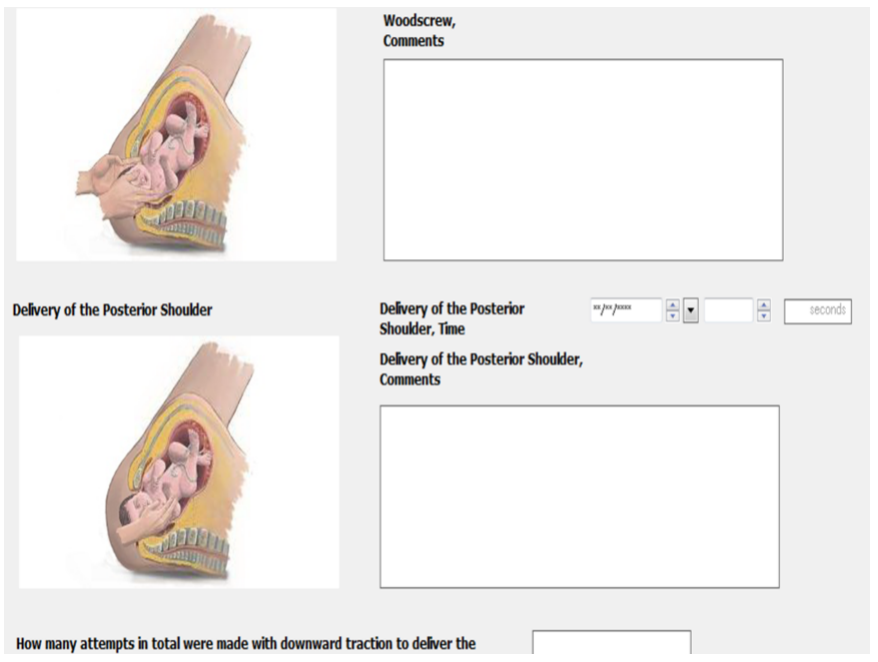
Example of educational documentation in MN-CMS—shoulder dystocia. MN-CMS: Maternal & Newborn Clinical Management System.

The MN-CMS has allowed clinical decisions support systems and educational links to be built into the system. These, for example, include visual color clues for abnormal values, the Irish Maternity Early Warning System, a sepsis alert algorithm, and reference text from national guidelines. There is also education-oriented documentation including that for shoulder dystocia (Figure 3) and sepsis. The increased functionality of the EHR allows staff to fully engage with it and be assisted in the care they provide through built-in clinical decision support systems. Key lessons from the optimization phase are outlined in Textbox 3.

**Optimization phase lessons learned.**

**Optimization phase lessons**

- Enhanced assessment of medication errors is essential towards adequate patient safety
- Ongoing staff training required to fully assist documentation efficiency
- Data Quality personnel need to be resourced early in the project and in post before go-live. A data strategy and framework need to be developed
- For relevant reports, data entry requirements need to be planned and configured in advance. Ensure staff understand their responsibility is data accuracy and completeness
- Engage with patient representatives through the go-live and informing them of potential disruptions
- All trainers and superusers should be kept involved and fully utilized
- Decision Support tools in the electronic health record assist staff in the care they provide

## Discussion

Digital technologies have transformed private industries such as banking, finance, transportation, navigation, internet search, retail, and now EHRs may bring the same revolutionary change to health care [18]. Globally, however, the overall implementation of EHRs has not been as successful as anticipated, and the benefits of EHRs have been slow to be realized [2]. It has been recognized that health information systems are needed, but key events in recent years have highlighted the need for this even further [21]. First, the global COVID-19 pandemic has accelerated the need for sharing of health data in the interest of public health [21], and, second, in Ireland the cyber security breach on the Irish Health Service Executive in May 2021 underlined the need for trust in data information security and the protection of health data [21]. The data generated from a national EHR have the potential to save lives around the world [22]. The Goldacre Review in the United Kingdom’s National Health Service has identified that data stored on tens of millions of people could have a huge potential on improving patient outcomes and saving lives if it is used and analyzed appropriately [22].

This paper has outlined the lesson learned in the Irish context for the development of the maternal and newborn EHR. The MN-CMS is an example of a clinician-led, patient-focused, change management project from its inception to implementation. The National Project team maintained patient care at the core of the development and implementation of this EHR and has provided valuable lessons for future EHR implementations. The importance of clinical leadership was integral throughout the process from planning, implementation, and optimization. Additionally, having the right people involved at the right stages of the project was essential. Challenges arose in terms of resourcing and often the same person was involved across multiple work streams such as testing, training, and change management [14]. The National Project team has since expanded and increased its resourcing since phase one. The key to ongoing support and optimization has been highlighted as essential to maintain the data quality and data input into the system. Regular data quality checks and ongoing training are essential post go-live. The key recommendations are summarized in Textbox 4.

**Key summary of recommendations.**

**Summary of recommendations**

- Clinical leadership is essential throughout the project
- Involving the right people at the right stages of the project; for example, clinical staff needed for workflow development and information and communication technology staff needed for hardware setup
- Adequately resourced team for various stages; for example, clinical, medications, testing, training, and support
- Strong governance structure and strict change control process
- Ongoing support, training, and optimization required
- Data quality surveillance and development of a data strategy and framework
- Always keep the patient at the center of the project

Following the phase one implementation, the MN-CMS now encompasses 40% of all births in the Republic of Ireland [10] and provides the foundation of a national EHR [14]. The MN-CMS generates approximately 50 reports per day, which guides both planning of care and epidemiological research and audit reporting. Reports generated include daily delivery lists, admission and discharge reports, and reports for the General Register Office to name but a few. The ongoing deployment of the MN-CMS is dependent on future funding and resources from the Irish Department of Health. Phase 2 has gained approval and will progress over the next 2 years incorporating all the tertiary centers in Irelands and 70% of births. Devin et al assessed the impact of the introduction of MN-CMS in 1 tertiary site by measuring task distribution of neonatal intensive care staff [23]. This assessment concluded that the EHR introduction did not redirect time away from direct patient care as other studies have reported [23]. Some EHR implementations have negatively redistributed time toward documentation and medication management and away from direct patient care [23]. The Irish Health Information Quality Authority proposes that effective engagement (professional and public), technical and operational, legislative frameworks, and governance structures are the cornerstone of informing policy on the collection, uses, and sharing of health data [21]. The MN-CMS has demonstrated these pillars in the EHR implementation.

The future of EHRs has an exciting future with new technologies such as vital signs to be automatically recorded into the chart, direct dictation to the record and even automated assistants that listen to the interactions between doctor and patient, and from verbal cues record the information in the examination room [18]. Interactive patient portals will allow patients not only to review their data (ensuring accuracy) but also to input information prior to consultations and assist self-care as part of chronic disease management [2]. Following a public engagement in the Republic of Ireland, 82% of people want access to their own health information [24].

The volume of data contained in an EHR has significant potential to positively influence all of our lives if used correctly and adhering to all data protection and legislation [2,22]. The potential to use data may be the greatest effect on health care and will come from data science using the information available in the EHR [2]. Data are the core of improvements in health care, and with the correct and appropriate analysis improvements can be realized in quality, safety, and cost-effectiveness of care across the entire health service [22]. The governance of data, however, is key to the continued success of the development of the EHR. Patients need to be informed of how their data are used, why they are used in a particular way, and how that can improve care and conditions for themselves and others [2]. They also need to know their data are respected and entered correctly based on European General Data Protection Guidelines [17]. Academic institutions have realized the importance of health informatics and the importance of data retrieval, and they are now providing modules at the undergraduate level to prepare medical professionals [25]. The Goldacre review recommends the introduction of trusted shared data platforms to rapidly capitalize on skills and data usage [22]. The introduction of new analysts, academics, and innovators can provide well-curated data and accessible technical documentation to improve patient outcomes [22]. The potential value of data science is well recognized in the ICT world; tech guru, Vinod Khosla, has suggested that “*In the next 10 years data science will do more for medicine than all the biological sciences combined*” [26]. It is likely that big data will assist us answer questions about the best care that would not be possible if we await randomized trial approaches [2]. Getting to this latter point in development requires significant work in the planning and development of the EHR, the change management to move from a paper environment, and the need for ongoing training and optimization to assist staff interaction with the system thereby enhancing the data quality that will allow big data output to assist care. The change management process in moving to an EHR environment needs to be patient centered, clinician led, and ICT supported. Our experience shows some of the lessons we have learned.

### Conclusions

This paper has outlined the implementation of a national EHR for mothers and babies in Ireland. The experiences and lessons learned from the national project team have been explored and summarized. Key recommendations are that clinical leadership and strong governance are essential as well as having an adequately resourced team. Ongoing training and optimization are necessary, and data quality surveillance can lead to more accurate reporting from the system. The potential of accurate cumulative data from EHRs can have a positive influence on population health through appropriate analysis. Keeping the patient at the center of the project was always paramount. The MN-CMS has been successfully implemented into 4 maternity units in Ireland and encompasses 40% of births with a plan to expand further in the future.

## Abbreviations

EHR: electronic health record
ICT: information and communication technology
MN-CMS: Maternal & Newborn Clinical Management System
NICU: neonatal intensive care unit
